# A community-based cross-sectional study for relationship of frequency of vegetables intake and osteoporosis in a Chinese postmenopausal women sample

**DOI:** 10.1186/s12905-016-0307-5

**Published:** 2016-06-03

**Authors:** Najia Liu, Fangfang Zeng, Keqin Zhang, Zihui Tang

**Affiliations:** Department of Endocrinology and Metabolism, Shanghai Tongji Hospital, Tongji University School of Medicine, Shanghai, 200065 China; Department of Endocrinology and Metabolism, Fudan University Huashan Hospital, Shanghai, China

**Keywords:** Frequency, Vegetables intake, Osteoporosis, Chinese postmenopausal women, Association

## Abstract

**Background:**

The main purpose of this study was to explore the associations between frequency of vegetables intake and osteoporosis (OP) in Chinese postmenopausal women.

**Methods:**

We conducted a large-scale, community-based, cross-sectional study to investigate the associations by using self-report questionnaire to access frequency of vegetables intake. The total of 1903 participants was available to data analysis in this study. Multiple regression models to include frequency of vegetables variable were performed to investigate the relationships for OP, after controlling for confounding factors.

**Results:**

Multiple regression analysis indicated that the frequency of vegetables intake was independently and significantly associated with OP (*P* < 0.1 for model 1 and model 2). The postmenopausal women with high frequency of vegetables intake had a higher prevalence of OP.

**Conclusion:**

The findings indicated that frequency of vegetables intake was independently and significantly associated with OP. The prevalence of OP was more frequent in Chinese postmenopausal women preferring vegetables food habits.

**Trial registration:**

(ClinicalTrials.gov Identifier: NCT02451397; date of registration: 2015-05-28).

## Background

Osteoporosis (OP), an important and complex health concern for post-menopausal women, is a systemic skeletal disease that is characterized by the increased risk of fragility fractures and reduced bone mineral density (BMD). Osteoporosis can affect men and women of all ages, but is more common among post-menopausal women [1]. According to the available epidemiological data, in China, the prevalence of osteoporosis is estimated at 5.5 to 15.5 % for men and 11.8 to 24.5 % for post-menopausal women [2]. Osteoporosis has become an important public health problem in China due to increased life expectancy and lifestyle changes [3].

Although bone density is largely determined by heredity and genetics, a number of lifestyle factors also play a role in the development or prevention of osteoporosis, including nutritional factors such as intake of calcium, protein, dairy foods, fruits and vegetables and vitamin D, and behavioral factors such as physical activity, smoking, and alcohol consumption, through their effects on bone development during growing years and the rate of bone loss in later life [4]. In previous studies, most of the attention has been directed towards the roles of calcium, dairy foods, and vitamin D in bone health [5–7]. In addition to calcium and vitamin D, a wide range of nutritional supplements have been recommended to improve low bone density, but the evidence of their benefit is limited [8–10]. The absence of this evidence may mean that more study is required.

It was appropriate and convenient for the current research project to conduct a large-scale study to evaluate risk factors for common diseases using a self-reported questionnaire. A subjective self-report questionnaire can accurately reflect the number of vegetables consumed [11]. Our previous association analyses for osteoporosis in Chinese postmenopausal women showed the relationships among meat consumption, coffee consumption, coronary artery disease and this outcome [12–14].

Several studies have demonstrated that diets rich in vegetables result in lower dietary acid loads due to their high concentrations of potassium and magnesium (an acid environment is known to stimulate osteoclasts and reduce osteoblast activity), and thus promote a positive calcium balance [9, 15, 16]. However, one study reported that BMD and calcium intake were not significantly different in vegetarian and non-vegetarian Taiwanese adults [15]. Therefore, more studies need to be conducted on more populations. To the best of our knowledge, little is known of the association between the frequency of vegetable intake and OP in the Chinese population. The main purpose of this study was to evaluate the extent to which the frequency of vegetable intake was associated with OP among the Chinese post-menopausal population using self-reported questionnaire methods applied to a large-scale sample.

## Methods

### Study population

As we mentioned earlier [13, 14], a risk-factor study for OP was performed in a random sample of the Chinese population. Participants were recruited from rural and urban communities in Shanghai. Participants aged 30–90 years were included in this study. More than 2000 postmenopausal women were invited to a screening visit between 2011 and 2014. Exclusion criteria were described earlier [13, 14], some participants with chronic diseases and conditions that might potentially affect bone mass, structure, or metabolism were excluded. Briefly, the exclusion criteria were as follows: a history of 1) serious residual effects of cerebral vascular disease; 2) serious chronic renal disease (Glomerular filtration rate - GFR < 30 mL/min/1.73 m^2^); 3) serious chronic liver disease or alcoholism; 4) significant chronic lung disease; 5) corticosteroid therapy at pharmacologic levels; 6) evidence of other metabolic or inherited bone disease, such as hyper- or hypoparathyroidism, Paget disease, osteomalacia, or osteogenesis imperfecta; 7) recent (within the past year) major gastrointestinal disease, such as peptic ulcer, malabsorption, chronic ulcerative colitis, regional enteritis, or significant chronic diarrhea; 8) Cushing syndrome; 9) hyperthyroidism; and 10) any neurologic or musculoskeletal condition that would be a non-genetic cause of low bone mass. A total of 1903 participants was available for data analysis in this study.

### Ethics statement

This study was reviewed and received ethical approval from the Ethics Committee at the Shanghai Tongji Hosptial. Permission to conduct the study was granted by the Shanghai Tongji Hosptial. Written informed consent was obtained from all study participants.

### Data collection

All study subjects underwent complete clinical baseline characteristics evaluation. As we mentioned earlier [13, 14], a physical examination and response to a structured, nurse-assisted, self-administrated questionnaire were conducted to collect information on age, gender, residential region, visit date, family history, lifestyle, dietary habits, physical activity level during leisure time, use of vitamins and medications, smoking, alcohol consumption, and self-reported medical history. Body weight and height were measured according to a standard protocol. The definitions of smoking and alcohol consumption, regular exercise, education level, HTN, and DM were described earlier [13, 14].

Dietary habits, including consumption of meat and potato food and vegetables was evaluated by a semi-quantitative food frequency questionnaire (group 1: once or twice per week, group 2: once per 2 days, and group 3: always). To determine frequency of fish food preference, the participants were asked, “How often you eat vegetables?” The possible answers were: “once or twice per week,” “once per 2 day,” or “always,” and the answers were taken as a subjective assessment. To answer the question, the participants were required to decide two issues based on their impressions: 1) whether or not the consumed vegetables were actually vegetables; and 2) the frequency with which they consumed vegetables. Generally, the way used medium cook to prepare vegetables in China.

### The study outcomes

As we mentioned earlier [13, 14], the bone mineral density (BMD g/cm^2^) was measured at calcaneus by standardized quantitative ultrasound (QUS, Hologic Inc., Bedford, MA, USA) utilizing T-scores based on WHO criteria, which were obtained from the automated equipment. T-score refers to the ratio between patient’s BMD and that of young adult population of same sex and ethnicity. T-score of > −1 was taken as normal, between −1 and −2.5 osteopenic and < −2.5 as osteoporotic.

### Statistical analysis

Continuous variables were analyzed to determine whether they followed normal distributions, using the Kolmogorov-Smirnov Test. Variables that were not normally distributed were log-transformed to approximate a normal distribution for analysis. Results are described as mean ± SD or median, unless stated otherwise. Differences in variables among subjects grouped by frequency of meat food intake were determined by one way analysis of variance. Among groups, differences in properties were detected by *χ*^2^ analysis.

For the associations analysis, there model have been developed. In model 1, frequency of vegetables intake were categorized by group 1: once or twice per week, group 2: once per 2 days, and group 3: always. In model 2: frequency of vegetables intake were categorized by low frequency and high frequency groups. Univariate regression analysis was performed to determine variables associated with OP, and to estimate confounding factors possibly disturbing the relation of frequency of vegetables intake to OP. Tests were two-sided, and a *p*-value of < 0.05 was considered significant. Multivariable regression (MR) was performed to control potential confounding factors and determine the independent contribution of variables to OP. Under MR models, tests were two-sided, and a *p*-value of < 0.1 was considered significant.

Results were analyzed using the Statistical Package for Social Sciences for Windows, version 16.0 (SPSS, Chicago, IL, USA). Odds ratios (OR) with 95 % confidence intervals (CI) were calculated for the relative risk of frequency of fish food intake with the outcome of OP.

## Results

### Clinical characteristics of subjects

The clinical baseline characteristics of the 1903 Chinese postmenopausal women were described earlier [13, 14] (Table 1). In the total sample, the mean age was 62.39 years. The minority proportions of subjects having current smoking and alcohol habits were reported (0.79 and 2.10 % for smoking and drink intake, respectively). The prevalence of HTN, coronary artery disease (CAD), DM and Rheumatoid arthritis (RA) were 45.51, 10.04, 11.49, and 5.72 %, respectively. There were significant differences in age and exercise among groups according to frequency of vegetables intake (*P* value <0.05 for all). An average T-score of −1.86 was reported and the prevalence of OP was 28.32 % in our study sample. Significant differences in the prevalence of OP among the three groups were reported (*P* value =0.015 for the prevalence of OP).

**Table 1:** Baseline characteristics of subjects

Variable	Total sample	Frequency of vegetables intake*			*P*-value
		Group 1	Group 2	Group 3	
Demographical information					
N	1903	196	58	1649	-
Age	62.39 ± 8.98	60.4 ± 9.05	60.28 ± 9.35	62.7 ± 8.92	0.001
Height	156.35 ± 5.71	153.73 ± 5.37	158.39 ± 6.55	156.51 ± 5.59	0.173
Weight	58.85 ± 8.47	56.86 ± 7.34	56.67 ± 7.75	59.47 ± 8.73	0.463
Lifestyle					
Education	254(13.35 %)	22(11.22 %)	6(10.34 %)	226(13.71 %)	0.179
Exercise	1362(71.57 %)	179(91.33 %)	49(84.48 %)	1134(68.77 %)	<0.001
Smoking	15(0.79 %)	4(2.05 %)	0(0 %)	11(0.67 %)	0.253
Alcohol intake	40(2.1 %)	6(3.06 %)	1(1.72 %)	33(2 %)	0.099
Medical history					
HTN	835(44.51 %)	84(43.75 %)	21(37.5 %)	730(44.84 %)	0.540
CAD	183(10.04 %)	15(7.85 %)	4(7.14 %)	164(10.41 %)	0.414
DM	214(11.49 %)	21(11.35 %)	6(11.54 %)	187(11.5 %)	0.998
RA	105(5.72 %)	7(3.7 %)	2(3.85 %)	96(6.02 %)	0.363
Therapy history					
Vitamin C	246(12.93 %)	16(8.16 %)	6(10.34 %)	224(13.58 %)	0.277
Vitamin D	78(4.1 %)	2(1.02 %)	3(5.17 %)	73(4.43 %)	0.239
Dietary habit					
Meat	848(44.56 %)	99(50.51 %)	29(50 %)	720(43.66 %)	0.133
Potato	264(13.87 %)	21(10.71 %)	20(34.48 %)	223(13.52 %)	<0.001
Oil	19.13 ± 9.05	16.06 ± 10.13	23.03 ± 11.95	19.36 ± 8.7	<0.001
Outcome					
T-score	−1.86 ± 0.74	−1.79 ± 0.74	−1.78 ± 0.75	−1.88 ± 0.74	0.209
OP	539(28.32 %)	39(19.9 %)	14(24.14 %)	486(29.47 %)	0.015

Note: *Frequency of vegetables intake were categorized by group 1: once or twice per week, group 2: once per 2 days, and group 3: always; *HTN* hypertension, *CAD* coronary artery disease, *DM* diabetes mellitus, *RA* Rheumatoid arthritis, *OP* Osteoporosis

### Univariate analysis for OP

**Fig. 1:**
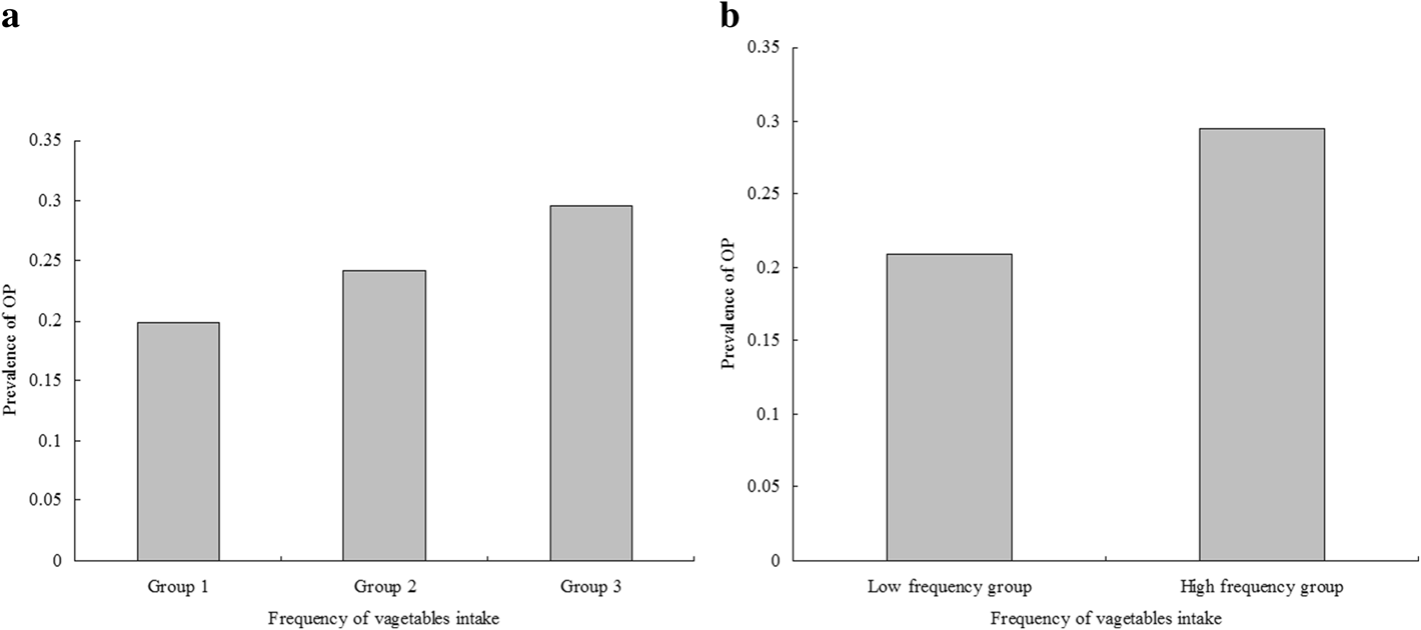
Comparison of prevalence of osteoporosis among groups according to frequency of vegetables intake in Chinese postmenopausal women. **a** The results of comparison of prevalence of osteoporosis among groups according to Model 1 (Model 1: frequency of vegetables intake were categorized by group 1: once or twice per week, group 2: once per 2 days, and group 3: always). The prevalence of osteoporosis was 19.90, 24.14 and 29.47 % in the three groups, respectively. There were significantly differences among the three groups (*P* value = 0.015). **b** The results of comparison of prevalence of osteoporosis between groups according to Model 2 (Model 2: frequency of vegetables intake were categorized by low frequency and high frequency groups). The prevalence of osteoporosis was 20.87 and 29.47 % between the two groups, respectively. There were significantly differences between the two groups (*P* value = 0.005)

Univariate logistic analyses were performed to evaluate associations with OP. As we mentioned earlier [13, 14], the results indicate that age, education, HTN, CAD, RA, Vitamin C and frequency of vegetables intake were significantly associated with OP (*P* value < 0.05 for all, Table 2). The comparison of prevalence of OP among groups according to model 1 reported that the prevalence of OP was 19.90, 24.14 and 29.47 % in the three groups, respectively (Fig. 1a). There were significant differences among the three groups (*P* value = 0.015). Significant differences among groups according to model 2 were also reported (Fig. 1b, *P* value = 0.005 for model 2). Univariate analysis demonstrates a positive correlation between frequency of vegetables intake and OP.

**Table 2:** Univariate logistic regression analysis for associations among variables and osteoporosis

Variable	*β*	S.E.	*P* value	OR	95.0 % CI
Age	0.099	0.007	<0.001	1.104	1.09–1.119
Height	0.008	0.05	0.871	1.008	0.914–1.112
Weight	0.005	0.033	0.886	1.005	0.941–1.073
Education	−0.24	0.044	<0.001	0.787	0.722–0.858
Exercise	−0.236	0.111	0.033	0.790	0.636–0.981
Smoking	0.03	0.276	0.913	1.031	0.601–1.769
Drink	−0.193	0.192	0.315	0.824	0.565–1.202
HTN	0.31	0.103	0.003	1.364	1.114–1.668
CAD	0.498	0.162	0.002	1.646	1.198–2.26
DM	0.178	0.157	0.255	1.195	0.879–1.625
RA	0.48	0.208	0.021	1.616	1.075–2.429
Vitamin C	0.314	0.145	0.030	1.369	1.03–1.817
Vitamin D	0.492	0.237	0.038	1.636	1.028–2.603
Frequency of vegetable intake	0.261	0.091	0.004	1.298	1.087–1.551

Note: *HTN* hypertension, *CAD* coronary artery disease, *DM* diabetes mellitus, *RA* Rheumatoid arthritis

### Multiple variable analyses for OP

Multivariate logistic regression analyses were employed to evaluate the association between frequency of vegetables intake and the OP outcome. After adjustment for relevant potential confounding factors, the multivariate logistic regression analyses detected significant associations (*p*-value = 0.082 for model 1; and *p*-value = 0.095 for model 2, Table 3). In participants with high frequency of vegetables intake, the OR for OP was 1.341 (95 % CI: 0.951–1.891).

**Table 3:** Multiple variables logistic regression analysis for associations between frequency of vegetables intake and osteoporosis

Model	Variable	*β*	S.E.	*P* value	OR	95 % CI
Model 1	Frequency of vegetables intake	0.172	0.099	0.082	1.188	0.979–1.443
Model 2	Frequency of vegetables intake	0.293	0.175	0.095	1.341	0.951–1.891

Note: Model 1: frequency of vegetables intake were categorized by group 1: once or twice per week, group 2: once per 2 days, and group 3: always; Model 2: frequency of vegetable intake were categorized by low frequency and high frequency groups; and all models adjusted for age, smoking, alcohol intake, education, exercise, medical and therapy history

## Discussion and conclusion

Osteoporosis has recently been recognized as a major public health problem by several governments and health care providers [17]. Menopause is associated with a reduction in estrogen secretion in women, resulting in decreased bone density that can lead to severe osteoporosis [18]. A large-scale, community-based, cross-sectional study was conducted to estimate the association between high frequency vegetable intake and OP in a sample of Chinese post-menopausal women. A subjective self-reported questionnaire was used to evaluate vegetable intake; it was found to be suitable for this large-scale study because of the convenience it provided in collecting independent variables. In this investigation, BMD was evaluated using QUS, which has many advantages in assessing osteoporosis: the modality is small, no ionizing radiation is involved, measurements can be made quickly and easily, and the cost of the device is low compared with DXA and quantitative computed tomography devices.

When studying diseases, including OP, physicians have been trained to better understand the effect of modifiable risk factors on outcomes so that we can control them in order to efficiently decrease the overall risk of a negative outcome. Frequency of vegetable intake may be considered one of these modifiable risk factors, and along with age, education, HTN, CAD, RA, and vitamin C intake, it was found to be significantly associated with OP. These results are consistent with the results of nationwide epidemiological studies; we found that frequent intake of vegetables is associated with a higher risk of osteoporosis among post-menopausal Chinese women using a multiple-variable logistic regression analysis adjusted for age, smoking, alcohol intake, education, exercise, and medical and therapy history. To date, numerous studies have shown that calcium intake is associated with bone mineral density [5, 6, 19]. Inadequate calcium intake influences calcium-regulating hormones, causing increased rates of bone remodeling without concomitant redeposition and leading to increased fracture risk [20]. Dairy products are an important dietary source of calcium in many populations, and the benefits of dietary calcium from dairy sources on bone metabolism are markedly greater than those of nondairy sources or calcium supplements [21, 22]. Numerous studies have assessed the relationship between bone density and protein intake. While a high-protein diet is known to aid skeletal growth during the growth period, and a positive correlation between bone density and protein intake has been previously reported, it has also been shown to accelerate the degeneration of renal structure and function, resulting in increased urinary elimination of calcium during aging [23]. This is critical to our study because dark green vegetables are a viable source of dietary calcium [24, 25], although the calcium they contain has low bioavailability compared to the calcium in dairy sources. While there is evidence suggesting that adequate protein intake and higher intake of fruits and vegetables are beneficial to bone health [4], our study provided evidence that a high frequency of vegetable intake could have adverse effects on osteoporosis among Chinese post-menopausal women. This conclusion was inconsistent with those of several previous studies, and it caused us to reconsider the role of vegetables in the prevention of osteoporosis among our sample.

In the past, vegetables have been considered important sources of calcium, especially for people who do not often drink milk. Calcium in dairy products has high bioavailability, and thus is more easily absorbed in the intestine than calcium from nondairy products, like dark green vegetables. Collard greens, kale, spinach, mustard leaves, turnip leaves, cabbage, broccoli, potatoes, pulses, and soybeans are common dietary sources of calcium, but as mentioned above, the calcium they contain has low bioavailability. On the contrary, other kinds of vegetables, particularly those containing calcium oxalic acid, phytic acid and tannic acid, combine with the calcium in food and form insoluble calcium salt, so that the body cannot absorb calcium. This might explain why a high frequency of vegetable intake could increase the prevalence of osteoporosis among some Chinese post-menopausal women. Specifically, the majority of vegetables they consumed may have been from those species mentioned above. Evidence indicates that the species of vegetables play a crucial role in the prevention of osteoporosis.

The limitations of this study were several and all warrant mention. Firstly, case–control studies like this one are prone to selection bias. A cross-sectional study for association analysis also requires a larger sample size and more geographic representation than was the case in this study. Besides, a subjective self-reported questionnaire was used to estimate vegetable intake for convenience in a large-scale cross-sectional study. Additionally, the questionnaire needed to include a question similar to: ‘What kind of vegetables do you consume?’ in order to determine whether the vegetables that subjects consumed were of the beneficial type. Thirdly, for data analysis, group 3 has been formed with a higher number of members than groups 1 and 2. Patients with RA have been included. “calcium intake” has not been evaluated in the diet questionnaire. These confounding factors may influence our results. Finally, the association results in this study need to be verified by follow-up studies using objective measures to evaluate actual vegetable intake.

Our findings suggested that frequency of vegetable intake with medium cooked was independently and significantly associated with osteoporosis. The prevalence of osteoporosis was higher in Chinese post-menopausal women who frequently consumed vegetables. According to our findings, a change in preference or dietary habits might be beneficial in the prevention of osteoporosis in Chinese postmenopausal women.

### Abbreviations

BMD, bone mineral density; BMI, body mass index; BM-MNC, bone marrow-derived mononuclear cell; CAD, coronary artery disease; CI, confidence intervals; DM, diabetes; DXA, dual-energy X-ray; GFR, glomerular filtration rate; HTN, hypertension; OP, osteoporosis; OR, odds ratios; QUS, quantitative ultrasound; RA, rheumatoid arthritis.
